# Ring artifact and Poisson noise attenuation via volumetric multiscale nonlocal collaborative filtering of spatially correlated noise

**DOI:** 10.1107/S1600577522002739

**Published:** 2022-04-21

**Authors:** Ymir Mäkinen, Stefano Marchesini, Alessandro Foi

**Affiliations:** a Tampere University, Finland; b SLAC National Accelerator Laboratory, 2575 Sand Hill Road, Menlo Park, CA 94025, USA

**Keywords:** BM4D, noise, image processing, tomography, artifact

## Abstract

X-ray micro-tomography systems often suffer from high levels of noise. In particular, severe ring artifacts are common in reconstructed images, caused by defects in the detector, calibration errors, and fluctuations producing streak noise in the raw sinogram data. Furthermore, the projections commonly contain high levels of Poissonian noise arising from the photon-counting detector. This work presents a 3-D multiscale framework for streak attenuation through a purposely designed collaborative filtering of correlated noise in volumetric data. A distinct multiscale denoising step for attenuation of the Poissonian noise is further proposed. By utilizing the volumetric structure of the projection data, the proposed fully automatic procedure offers improved feature preservation compared with 2-D denoising and avoids artifacts which arise from individual filtering of sinograms.

## Abstract 2

Streaks and Poissonian noise in tomography data are attenuated using a new two-stage multiscale volumetric filtering framework where the degradations are modeled as correlated noise. The procedure is fully automatic, offers improved feature preservation, and can be further combined with regularized reconstructions to deliver state-of-the-art imaging quality.

## 1. Introduction

Computed tomography is commonly affected by streak noise in measured raw sinogram data (Jha *et al.*, 2013 ▸; Artul, 2013 ▸; Boas & Fleischmann, 2012 ▸), which can be caused by mis-calibration of detector linear response, beam fluctuations, beam hardening, or dusty or damaged scintillator screens (Haibel, 2008 ▸; Vidal *et al.*, 2005 ▸; Anas *et al.*, 2010 ▸). Streak noise in projections causes ring artifacts in reconstructed volumes, which present as centered circles or half-circles (Croton *et al.*, 2019 ▸). As the sinogram data are obtained through a photon-counting detector, the statistics of the measured raw data can be further modeled through a Poisson distribution, which may result in high levels of Poissonian noise, commonly attenuated within the reconstruction process through iterative approaches (Mohan *et al.*, 2014 ▸; Venkatakrishnan *et al.*, 2013 ▸).

Although ring artifacts can be reduced by scanning protocols (Pelt & Parkinson, 2018 ▸), high-quality scintillator screens and detectors, it is difficult to completely avoid them and therefore achieve highest-quality reconstruction solely by experimental measures, requiring algorithmic processing of the acquisitions.

Popular methods to reduce ring artifacts include wavelet-FFT filters (Münch *et al.*, 2009 ▸), combinations of polynomial smoothing filters and careful calibration of the detector response function (Vo *et al.*, 2018 ▸; Croton *et al.*, 2019 ▸), smoothing filters with segmentation in the tomogram domain (Massimi *et al.*, 2018 ▸), ring removal in the tomogram domain upon polar coordinates transformation (Sijbers & Postnov, 2004 ▸; Li *et al.*, 2021 ▸), and iterative algorithms (Paleo & Mirone, 2015 ▸) that combine regularized reconstruction with denoising. Recently, in Mäkinen *et al.* (2021 ▸) ▸, we proposed effective ring artifact attenuation through sinogram-domain collaborative filtering, presenting a multiscale architecture with a Block-matching and 3-D filtering (BM3D) image denoiser for correlated noise (Dabov *et al.*, 2008 ▸; Mäkinen *et al.*, 2020 ▸) at the core of the process. To the best of our knowledge, Mäkinen *et al.* (2021) ▸ offers state-of-the-art results in ring attenuation. In particular, it does not cause new artifacts around strong signal features, common to other popular ring removal algorithms. However, being based on a filter for 2-D data^1^, applied to individual sinograms, it may cause discontinuities across the third dimension.

In this work, we address both streak reduction and Poissonian noise removal from volumetric stacks of projections. The contribution of this work is threefold: (1) We propose a multiscale streak denoising framework for denoising of volumetric data. In particular, this framework can be seen as an extension of Mäkinen *et al.* (2021) ▸ for filtering of 3-D volumes. (2) After streak noise removal, and before reconstruction, we embed a distinct multiscale denoising step to attenuate the Poissonian noise component of the projections. This allows to apply the reconstruction process using milder regularization and improve the tradeoff between noise reduction and artifact suppression. (3) As a general-purpose algorithmic contribution, the filter used at the core of the multiscale denoising process is an improved version of the BM4D (Maggioni *et al.*, 2012 ▸) volumetric denoising algorithm. The included enhancements, discussed in Appendix *A* [App appa], allow the long-range noise correlation which characterizes the streaks to be dealt with.

The proposed filtering procedure for both streaks and Poissonian noise is fully automatic and includes self-calibration of the filtering strength. We demonstrate the denoising performance of the proposed approach on real data from the table-top Prisma XRM microCT at Sigray, and from the synchrotron-based microCT at the Advanced Photon Source (APS) in Argonne, available through Tomobank (De Carlo *et al.*, 2018 ▸).

## 2. Bright-field normalization

The following normalization of the raw projections and the streak model upon a logarithmic transformation follow that of Mäkinen *et al.* (2021) ▸.

The optical attenuation through the sample is determined experimentally via bright-field corrections through two separate acquisitions, the bright-field and the dark-field (Seibert *et al.*, 1998 ▸). The bright-field is obtained by the imaging procedure with no sample, and the dark-field is obtained with no beam; both are 2-D arrays the size of effective pixels of the detector. The Beer–Lambert law further relates the X-ray transform through the sample to the optical attenuation by a logarithmic transformation (Swinehart, 1962 ▸).

Hence, the raw projections *P*_raw_ are first normalized as 

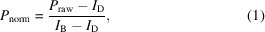

where *I*_D_ is the dark-field and *I*_B_ is the bright-field^2^, and then log-transformed as 

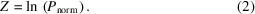




### 2.1. Noise model for normalized projections

Apart from possible completely defective detectors^3^ we treat the variation in detector response as normally distributed; as such, the streak noise will follow a normal distribution. Furthermore, we model the streak noise as locally stationary, meaning that the variance is presumed constant within the support of the denoising filter. Note that this does not mean that the noise is i.i.d. or white, as it is instead characterized by very long range correlation presenting as streaks.

As the data are obtained through a photon-counting detector, the statistics of the measured raw data can be further modeled through a Poisson distribution with nonstationary parameters after the bright-fielding.

Given the premises of normally distributed streak noise and Poissonian noise, noise in projections normalized by (1) can be formally written as 

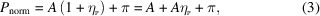

where *A* are the underlying noise-free projections, 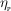 is the normally distributed streak noise component, and π is Poissonian noise which we model as white and zero-mean; all components of (3) are considered as 3-D arrays and multiplications are elementwise.

The natural logarithm of (2) acts as a variance-stabilizing transformation (VST) for the multiplicative noise component 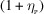 . Hence, we have 

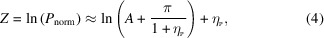

where the approximation comes from 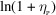 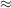 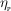 .

## 3. Correlated noise

The denoising is conducted in two steps. First, we aim to estimate the ‘streak-free’ projections 

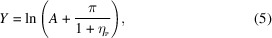

which are corrupted by white Poissonian noise. Then, as a separate denoising step, we consider the attenuation of the remaining noise originating from π.

Throughout this work, we will represent the volume to be filtered according to the correlated noise model presented in the following subsection. This model will assume different meaning at different parts of the algorithm. First, applying locally to the streaks as a type of long-range correlated noise; second, to the noise arising from the Poissonian component π.

### 3.1. Correlated noise model

We consider the noisy input 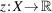 to be a combination of underlying data *y* and additive stationary spatially correlated noise η to be filtered, 

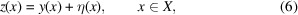

where 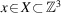 is the coordinate in the finite three-dimensional volumetric domain *X* and 

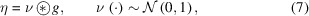

ν being zero-mean i.i.d. Gaussian noise with unit variance, and 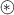 denoting 3-D convolution with the kernel *g*. The kernel *g* defines the spatial correlation of the noise as well as the noise strength, with 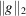 = 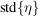 . An equivalent way of representing correlated noise is by its power spectral density (PSD) Ψ, 

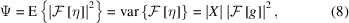

with 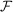 being the 3-D Fourier transform, and |*X*| denoting the cardinality (*i.e.* number of elements) of *X*. Equivalently, a kernel *g* satisfying (7)–(8) can be defined from Ψ as 

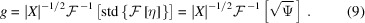




### 3.2. Estimation of noise standard deviation

When applying the above model to noisy data, it is essential to have knowledge of either the kernel *g* or, equivalently, the PSD Ψ, as they fully characterize the noise. Assuming *g* in (7) is known modulo a scaling factor ς from a known kernel *g*_*s*_, the noise estimation can be simplified to estimating ς. In particular, in order to model the streak and Poissonian noise components arising from the particular composition of noise given in (3), the kernels *g*_*s*_ should induce either very long range correlation or near white noise across each dimension *d*. The estimation procedure can be built as a direct extension of the one adopted by Mäkinen *et al.* (2021) ▸ to 3-D. To reduce the signal-to-noise ratio (SNR) to acquire a better noise estimate, we convolve *z* with a 3-D anisotropic kernel *g*_*d*_ that provides either low-pass or high-pass filtering along different dimensions; *g*_*d*_ is designed based on the noise statistics so that it preserves the noise component of interest while attenuating signal contrast. Specific instances are given in Section 4.1.1 and Section 5.3. One can then compute an estimate of the standard deviation of 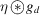 via its median absolute deviation (Hampel, 1974 ▸),

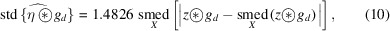

where smed denotes the sample median and the factor 1.4826 calibrates the estimate with respect to a normal distribution of the noise. As 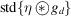 = 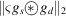 , an estimate 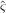 of ς can be obtained through 

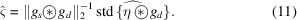




## 4. Multiscale streak filtering

In the following, we treat the first dimension of the stack of projections as the angular dimension, and the second and third as the horizontal and vertical displacement dimensions.

Because the streaks are inherently low-frequency with respect to the angle, they are filtered entirely at a *coarse angular scale*; for this task, we extend the multiscale procedure of Mäkinen *et al.* (2021) ▸. The main changes in the proposed procedure arise from replacing the one-dimensional binning operations along the displacement dimension with corresponding 2-D binning operators 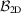 and 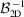 executed across both displacement dimensions. Furthermore, instead of using a direct 3-D extension of the 2-D streak PSD, we adjust the streak model to account for possible long correlation also along the displacement dimensions.

In detail, the multiscale streak attenuation procedure proceeds as follows. We begin by an angular binning 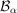 . The result of the angular binning 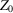 = 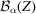 is binned *K* times through 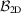 to obtain 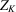 = 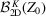 . The size of each binned volume is a quarter of the input size. Then, we process each scale in a coarse-to-fine fashion, where progressively for each 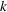 = 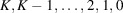 , we obtain an estimate 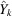 of 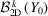 = 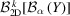 . We start by taking as noisy input 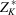 of BM4D the smallest binned volume *Z*_*K*_; in this way, we obtain from 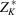 = 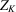 the coarsest estimate 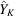 , which is taken as initialization for the following recursive steps executed for each scale 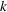 = 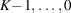 :

(1) Replace the coarser-scale components of *Z*_*k*_ by those of the estimate 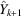 : 

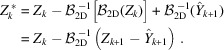




(2) Denoise 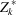 with BM4D to produce the estimate 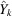 .

Finally, we replace the coarse angular components of the full-size stack *Z* with those from the finest scale estimate 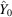 , 

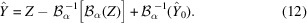




### 4.1. Multiscale noise model

For BM4D denoising, we regard 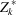 of each scale *k* as *z* of the model (6), as 

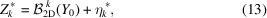

where 

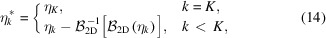

and 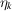 = 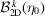 = 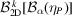 .

This definition for 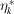 , 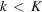 , follows from considering the coarser-scale estimate 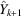 as perfectly denoised. Similar to (8), 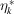 is treated as correlated noise with PSD, 

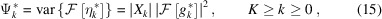

where 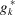 is a correlation kernel and |*X*_*k*_| is the pixel size of *Z*_*k*_. As per (9), the kernel 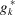 can be defined as 

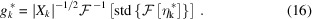




#### Adaptive parametric model of 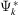


4.1.1.

We note that, in addition to the approximately white streak noise, the sinograms may contain streaks with very long range correlation across the displacement dimensions. As this correlation is aligned along the detector axis, it is not clearly observable in individual sinograms, but may create significant noise structure in the full volume. Hence, we approximate the streak noise η_0_ through three angularly constant streak noise components distinct in the displacement,

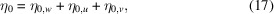

where η_0,*w*_ is streak noise white across both displacement dimensions, η_0,*u*_ is streak noise constant across horizontal displacement, and η_0,*v*_ is streak noise constant across vertical displacement.

**Figure 1:**
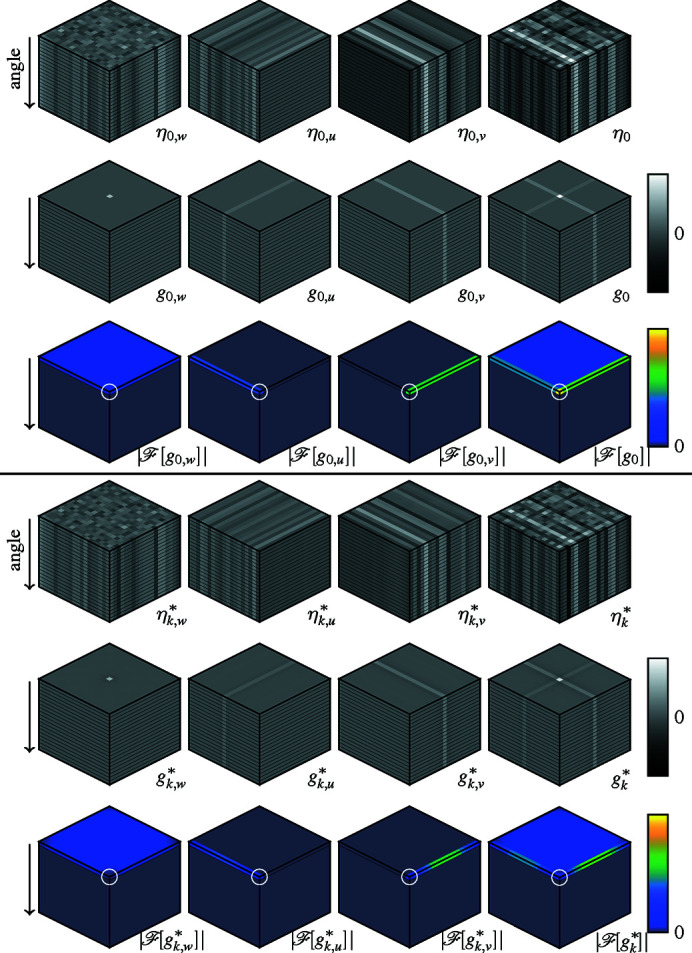
Top: example noise η_0,*p*_, the corresponding kernels *g*_0,*p*_ and the root PSD 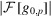 for each noise component in (17) with 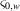 = 6, 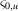 = 5, and 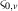 = 8, as well as example noise, kernel, and root PSD corresponding to the compound noise η_*k*_. Bottom: example noise, the corresponding kernels, and root PSD of the corresponding binning residuals 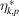 . For all visualizations, the angular dimension of the data is the vertical dimension in the figure. The DC corner of the Fourier spectra is marked by a circle. Note that all root PSDs are nonzero only on the angular DC plane, and the kernels and the noise consist of repeated planes across the angle.

Let us denote by 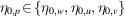 a noise component of η_0_. For each η_0,*p*_, we can define a respective scaled correlation kernel 

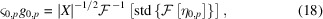

where 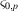 = 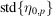 , and 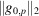 = 1. Example realizations, kernels, and PSDs for each of these components as well as η_*k*_ are shown in Fig. 1 ▸ (top).

We note that each 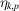 = 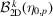 is characterized by a kernel 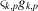 = 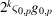 . In particular, this property arises from the noise structure of the corresponding components: as 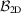 operates through summation and elimination of adjacent pixels, the operation preserves both noise whiteness and constant noise. The ratio 2^*k*^ follows from the summation along two dimensions, meaning that the variance of the coarser scale is four times that of the finer scale.

Disregarding the specific support size of their actual finite realizations, we can identify the stationary random fields as 

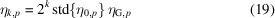

where η_G,*p*_ is noise characterized by *g*_*k*,*p*_, and hence 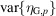 = 1. We can then express the residuals of any of the components η_0,*p*_ as 

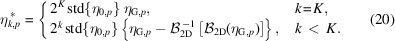

Then, 

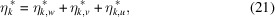

where the noise correlation kernel corresponding to a component 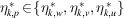 can be written with 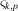 = 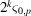 as 

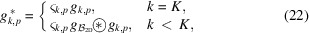

where 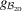 is a 2-D kernel across the displacement dimensions characterizing the residual from 2-D binning of white noise. Specifically, 

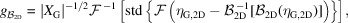

where η_G,2D_ is a two-dimensional white random field. The field size |*X*_G_| is included only for the normalization of the Fourier transform, and the formula holds for an arbitrary size.

Then, the PSD of 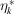 , 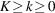 , can be written as 

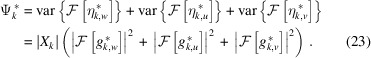

As any 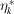 is constant in angle, 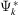 is non-zero only across the DC plane with respect to the angular dimension. Example realizations, kernels, and PSDs for the residual components are shown in Fig. 1 ▸ (bottom).

Although (23) allows for modeling of very long range correlation, the streak noise is likely to contain minor correlation along the displacement not accounted for by this model. To adapt to such deviations, we allow the scaling parameters 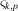 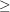 0 for each noise component to vary with each scale *k* by estimating them individually at each scale, effectively accounting for mild local correlation in the noise.

*Estimation of ς_*k*,*w*_, ς_*k*,*u*_, and ς_*k*,*v*_.* Based on (22) and (23), the PSD is completely determined by the values assumed by the three parameters ς_*k*,*w*_, ς_*k*,*u*_, and ς_*k*,*v*_ and the known kernels *g*_*k*,*p*_ and 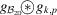 . To adaptively obtain the parameters, we begin by obtaining three noise variance estimates 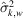 , 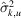 , and 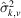 . For each estimate, we define a corresponding filtering kernel 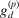 such that 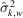 estimates the variance of high-frequency streaks, 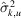 estimates the variance of horizontally low-frequency streaks, and 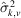 of vertically low-frequency streaks. For this purpose, we define ϕ_*d*_ as a 1-D Gaussian function along dimension *d*, and ψ_*d*_ as a 1-D high-pass kernel with Daubechies wavelet ‘db3’ of length 6 along *d*. Hence, convolution with ϕ_*d*_ realizes low-pass filtering, and ψ_*d*_ realizes a high-pass filter. Then, 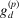 is realized as a tensor product of three one-dimensional kernels across the dimensions *d* chosen based on the noise statistics through that dimension,

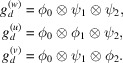

Specifically, with *m*_0_, *m*_1_, *m*_2_ as the pixel sizes of the three dimensions of 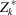 , ϕ_0_ is a 1-D Gaussian function along the angular dimension with standard deviation *m*_0_/8, and ϕ_1_ and ϕ_2_ are 1-D Gaussian functions along the two displacement dimensions with standard deviations of *m*_1_/12 and *m*_2_/12, respectively. Through these kernels, we obtain estimates of the three coefficients 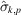 as described in (10) and (11) with *g*_*s*_ as either *g*_*k*,*p*_ ( 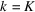 ) or 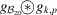 ( 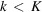 ).

We note that these three components do not directly correspond to ς_*k*,*w*_, ς_*k*,*u*_, and ς_*k*,*v*_, as the frequencies of the white streak component 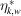 partly overlap with those of 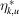 and 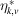 , *i.e.* 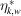 includes also some low-frequency streak components. In particular, we have 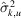 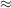 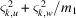 and 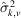 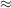 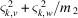 . To this end, we can formulate a simple non-negative least-squares optimization as 

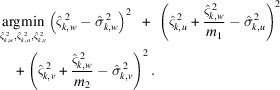

Finally, we construct the PSD through (22) and (23) with 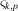 = 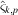 .

#### Nonstationarity of 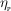


4.1.2.

Variance of the streak noise may differ across the sinogram due to changes in photon flux or noise in the bright-field. Thus, it may not be possible to denoise 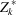 assuming an equal 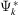 for all spatial positions without either oversmoothing or leaving noise artifacts in some areas. To adapt to nonstationarity, we further relax the streak model allowing the PSD to vary within each scale *k*. In particular, before noise estimation and denoising, we split 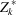 into overlapping, volumetric segments. We apply BM4D separately on each segment of 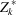 , using a PSD scaled by parameters estimated from the same segment, *i.e.* we consider each segment as a separate noisy volume *z* with a corresponding Ψ. After denoising, the segment estimates produced by BM4D are recombined with a windowing function to form the full estimate 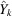 .

### 4.2. Attenuation of extreme streaks

We note that the projections often include several streaks caused by defects in the scintillator. These streaks can be far stronger than that reasonably produced by the distribution of 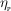 and therefore require a specific pre-processing. To this end, after the bright-fielding and log-transform and before the multiscale denoising procedure, we apply the simple extreme streak attenuation procedure as described by Mäkinen *et al.* (2021) ▸, which applies median filtering on extreme streak values detected through local polynomial fit of angular medians.

## 5. Poisson denoising

A filter for additive noise is not immediately applicable to the approximately white noise of 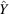 originating from the Poissonian component π. Firstly, the bright-fielding (1) introduces substantial spatial variability in the Poisson model. As a result, for a given optical attenuation, noise in bright-fielded projections can be stronger or weaker in different parts of the detector, for example around edges in cone-beam acquisition. Secondly, while the logarithm (2) effectively makes the streak noise additive, it also changes the typical affine-variance model of the Poissonian noise to a nonlinear one where the variance is not constant, but asymptotically inversely proportional to the mean. In order to model the noise in 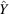 through (6), we take care of these two issues as follows.

### 5.1. Reducing nonstationarity induced by bright-fielding

The Poissonian noise component π originates from a counting process which takes place before bright-fielding (1), and specifically before the division by 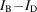 , which introduces a spatially variant scaling of the variances. To undo this scaling, we consider 

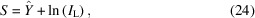

where 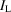 = 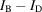 . Then, *S* can be treated as the log-scale version of a homogeneous Poissonian process; *S* is thus subject to signal-dependent noise where the variance of the noise can be expressed as a smooth nonnegative function of the underlying signal, 

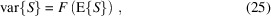

where the same *F* applies to each pixel. In particular, it can be shown that asymptotically for large flux 

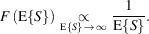




### 5.2. Stabilization of variance

**Figure 2:**
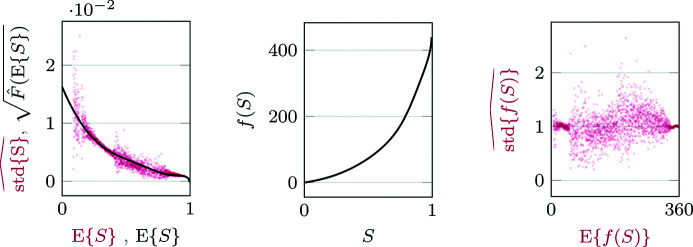
Illustration of the variance stabilization of *Fly*. Left: scatterplot of the sample standard deviation of *S* (red) as well as the square root of a polynomial approximation 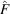 of *F* (black), computed on *S* scaled to the range [0, 1] as a function of its expectation using a fifth-degree polynomial variance model. Middle: the variance stabilizing transformation *f* for this 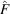 . Right: sample standard deviation of *f*(*S*) as a function of its expectation. The standard deviations and *f* are both computed using Foi (2009 ▸) and Azzari & Foi (2014 ▸). Every point of the red scatterplot corresponds to the sample mean and sample standard deviation of a narrow segment of the image; the dispersion of the scatterplot is due to the finite sample size of each segment.

To turn a model like (25) into (6) we again resort to the use of a VST. As large-flux asymptotics are irrelevant for denoising problems characterized by low signal-to-noise ratio, and to pragmatically accommodate for model uncertainties, we model *F* as a polynomial with arbitrary data-driven coefficients. The method (Foi, 2009 ▸; Azzari & Foi, 2014 ▸) simultaneously identifies the coefficients for an arbitrary signal-dependent noise model where the variance is a positive power of an unknown polynomial, and returns the associated variance stabilizing transformation *f* as well as the corresponding exact-unbiased inverse VST 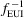 . An example of an estimated standard deviation function 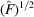 and the corresponding VST *f* are illustrated in Fig. 2 ▸, where the effectiveness of the stabilization can be deduced by the estimates of 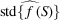 being scattered around 1.

### 5.3. Multiscale denoising of the stabilized Poisson noise

To avoid introducing structured artifacts that are present in the bright-field and dark-field images, we further consider a smoothed version 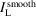 of the field component,

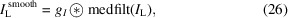

where *g*_*I*_ is a 2-D Gaussian kernel, and medfilt denotes a 2-D median filter. The median filter is adopted in order to remove extreme outliers (*e.g.* from broken pixels), and the convolution with the Gaussian ensures a smooth result. Then, 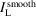 can be used for approximate correction for the bright-field induced nonstationarity with 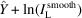 .

The stabilized noisy stack can then be written as 

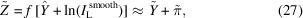

where 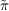 corresponds to the stabilized noise and 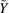 to the signal upon stabilization.

We consider π white, and assume the streak denoising procedure to remove all streak noise frequencies, including those of π. Hence, we treat 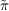 as missing the streak frequencies, *i.e.* with a PSD, 

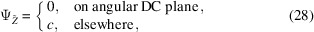

where *c* is a constant such that 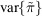 = 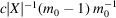 .

For multiscale denoising of the Poisson component, we define three-dimensional binning and debinning functions as 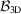 = 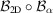 and 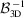 = 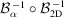 , and obtain *K*_Poi_ scales of binned noisy volumes as 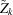 = 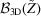 , 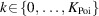 . Then, unlike the progressive denoising of the streaks, we begin by BM4D denoising of 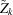 of each scale *k*; at each scale, we model the noise through a PSD of the form (28). This way, we obtain an initial estimate 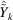 of the corresponding noise-free volume 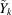 at each scale. Then, starting from 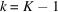 , we combine only the denoised volumes of each scale by recursively replacing the low-scale components of 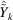 , 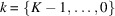 , by those of the lower scale,

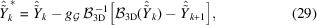

where 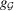 denotes a 3-D Gaussian kernel. Although the low frequencies are obviously denoised more effectively in the coarser scale, the higher frequencies of the coarser scale are commonly estimated worse than the respective estimate of the finer scale (Facciolo *et al.*, 2017 ▸). As such, 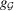 realizes a low-pass filtering which selects only low frequencies of the coarser estimate to be used in the full estimate.

To account for possible remaining nonstationarity and slight correlation of the noise, we perform the denoising in segments similar to as described in Section 4.1.2 for streak noise, and estimate a separate scaling parameter 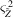 in construction of the PSD at each scale. In particular, we estimate 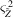 as described in Section 3.2 with 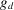 = 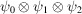 and 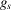 = 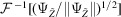 defining the unscaled noise correlation kernel, and finally construct the PSD through (28) with *c* = 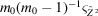 .

The final estimate of the underlying stack of projections can be obtained by applying 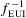 to the finest scale estimate 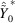 and then removing the field 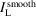 , 

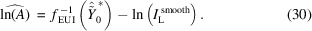

As (30) negates the field correction, we note that had we used the non-smooth field *I*_L_ in (27) [and respectively in (30)], any noise or spurious structures present in *I*_L_ could be introduced into 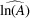 , as they might have been denoised by BM4D and hence not preserved in 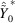 .

Upon variance stabilization, Poissonian data become asymptotically normal (Curtiss, 1943 ▸). Due to the additional Gaussianization induced by the binning and by the linear transformations operated by the filter, the assumption of normality in (7) can be adopted for denoising of the Poissonian component in this work even for low-count data.

**Figure 3:**
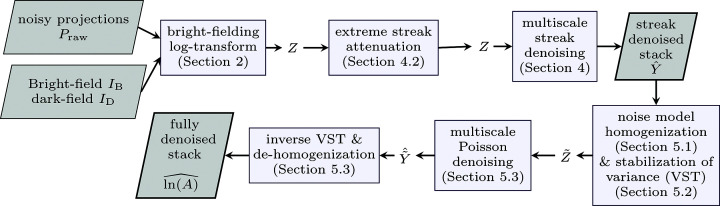
The full denoising process, requiring as inputs the noisy projections *P*_raw_ and the bright- and dark-fields *I*_B_, *I*_D_, (1) and producing as the output an estimate 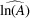 (30) of the underlying stack of projections ln(*A*) (3). As an intermediate output, an estimate 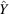 (12) of the streak-free yet noisy stack of projections *Y* (5) is also produced.

The full denoising process is shown in Fig. 3 ▸.

## 6. Experiments

We test our pipeline on synthetic data as well as two real acquisitions displaying ring artifacts and Poisson noise.

As a comparison, we show results for Mäkinen *et al.* (2021) ▸ available on PyPI as *bm3d-streak-removal*, the proposed algorithm embedding the conventional BM4D denoiser (Maggioni *et al.*, 2012 ▸), as well as two leading streak-removal procedures from the *tomopy* Python library (Gürsoy *et al.*, 2014 ▸): Münch *et al.* (2009) ▸ and Vo *et al.* (2018) ▸. In particular, for the latter we combine ‘Algorithm 3’, ‘Algorithm 5’, and ‘Algorithm 6’, which is demonstrated by Vo *et al.* (2018) ▸ to attenuate a variety of different streaks. These streak denoising algorithms are run with the default parameters provided by the software library. To evaluate the benefit of the proposed Poisson denoising procedure with reconstruction which includes further regularization of the data, we include experments with the iterative Total Variation (TV) reconstruction (Goldstein & Osher, 2009 ▸) of Marchesini *et al.* (2020) ▸.

For the synthetic experiments, we replicate the noise generation setup of Mäkinen *et al.* (2021) ▸ on a stack of projections ( 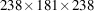 pixels) obtained from a 3-D BrainWeb phantom (Cocosco *et al.*, 1997 ▸) obtained through a padding and Radon transform upon a sign change and an exponential transformation. Specifically, we regard this stack as the underlying projections *A* and generate noise according to (3) with *g* as a constant of size 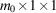 (equal to *g*_0,*w*_ of Fig. 1 ▸). To obtain streak noise of different strengths, the streak noise component 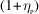 is generated with 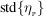 = 0.005, 0.01, 0.02, 0.05. Next, to generate noisy measurements with different SNR levels for the Poisson component, we separately scale *A* to the ranges [2560, 5120] (higher SNR), [1280, 2560], and [640, 1280] (lower SNR) and generate a Poisson variate with mean and variance 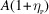 , thus defining the Poissonian noise π as the difference between this Poisson variate and 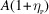 . Furthermore, we include experiments with π = 0 (infinite SNR), thus resulting in a total of 16 combinations of Poisson and streak noise strengths. We do not simulate extreme streaks or the bright-fielding ( 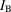 = 1 and 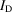 = 0). For the streak removal, we consider 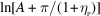 as the streak-free yet noisy stack *Y*.

**Figure 4:**
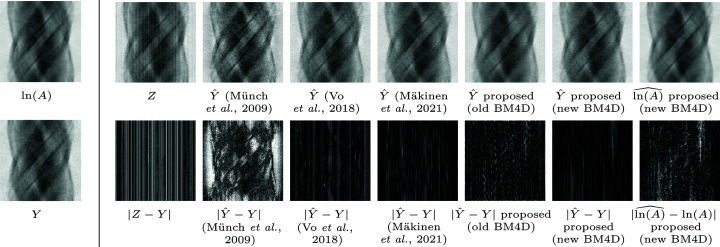
Denoising of the 3-D BrainWeb phantom with noise as in (3) with 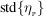 = 0.02 and signal peak 2560, displaying a single sinogram extracted from the stack. Left: a sinogram of ln(*A*) (3) and the corresponding streak-free but noisy sinogram of *Y* (5). Right: on top, noisy sinogram *Z* (4) and the comparison of estimates: 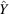 for Münch *et al.* (2009) ▸, Vo *et al.* (2018) ▸, Mäkinen *et al.* (2021) ▸, the proposed framework using old BM4D and using improved BM4D, and 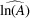 with the proposed framework and new BM4D. Below, corresponding estimation errors. Note that the errors 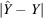 show only the effectiveness of streak removal, as the compared algorithms are designed for streak attenuation, whereas 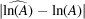 shows the complete denoising error, scaled separately. Notably, the proposed algorithm offers superior performance, but only when embedded with the improved BM4D.

The results of the phantom experiments^4^ for streak attenuation are collected in Table 1 ▸, and, for full denoising, evaluating the reconstructed volumes, in Table 2 ▸ using iterative regularized TV reconstruction with optimized regularization parameter strength *r*. The experiments for both streak and Poisson denoising are illustrated in Figs. 4 ▸ and 5 ▸. All reconstructions are performed upon a sign change.

**Figure 6:**
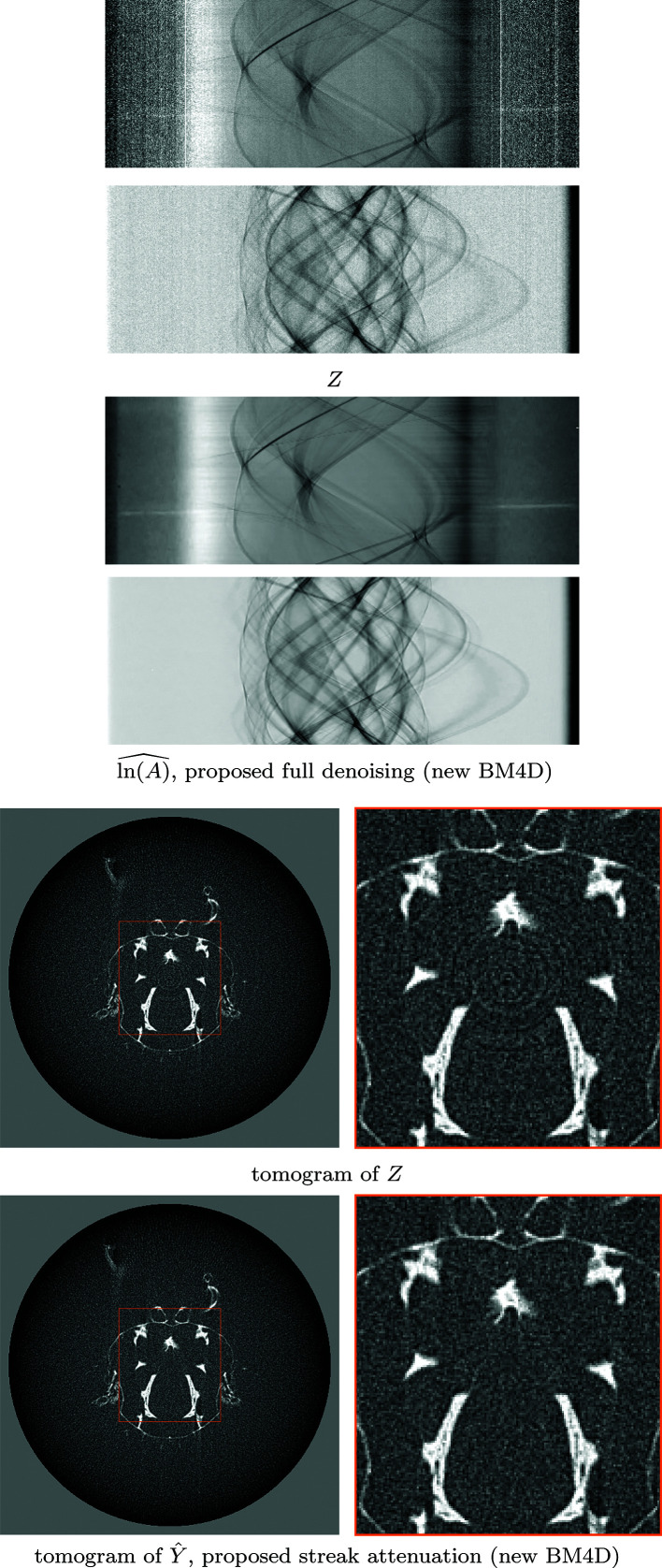
Denoising of the stack of projections of *Fly*, showing two sinograms of the noisy stack of projections *Z* (4) and the corresponding estimates 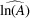 of the underlying stack of projections ln(*A*) (3) obtained with the proposed framework (top), and the tomograms of the second sinogram (bottom), obtained with filtered back-projection using cone-beam geometry (Feldkamp *et al.*, 1984 ▸), for both the noisy data *Z* and the proposed estimate 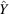 of the streak-free projections *Y* (5). The tomogram for 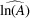 is shown in Fig. 8 ▸. The first sinogram shows significant model nonstationarity in both streaks and the Poissonian component due to the bright-fielding.

**Figure 7:**
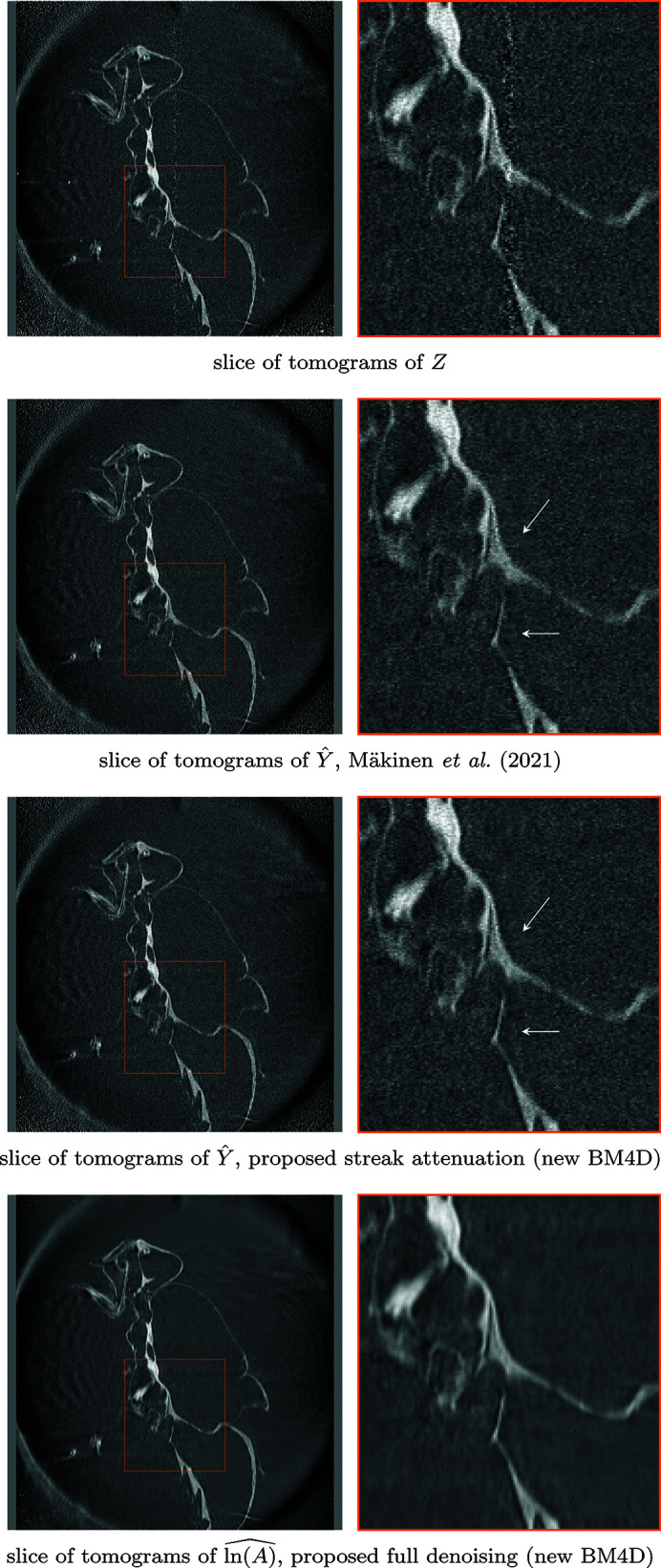
Comparison of resulting stack of tomograms after different denoising procedures on *Fly*, each obtained with filtered back-projection, where individual tomograms are horizontal lines. Displayed are slices of tomograms, reconstructed from, top-to-bottom: noisy projections *Z* (4), estimates 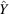 of the streak-free projections *Y* (5) denoised with Mäkinen *et al.* (2021) ▸, 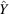 from the proposed streak denoising, and the result of the proposed full denoising 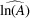 . Note the horizontal ‘streaks’ in the estimate produced by Mäkinen *et al.* (2021) ▸, which arise from differences in estimates for consecutive slices; the proposed method is not prone to such artifacts, as it considers the full stack of projections in the denoising.

**Figure 8:**
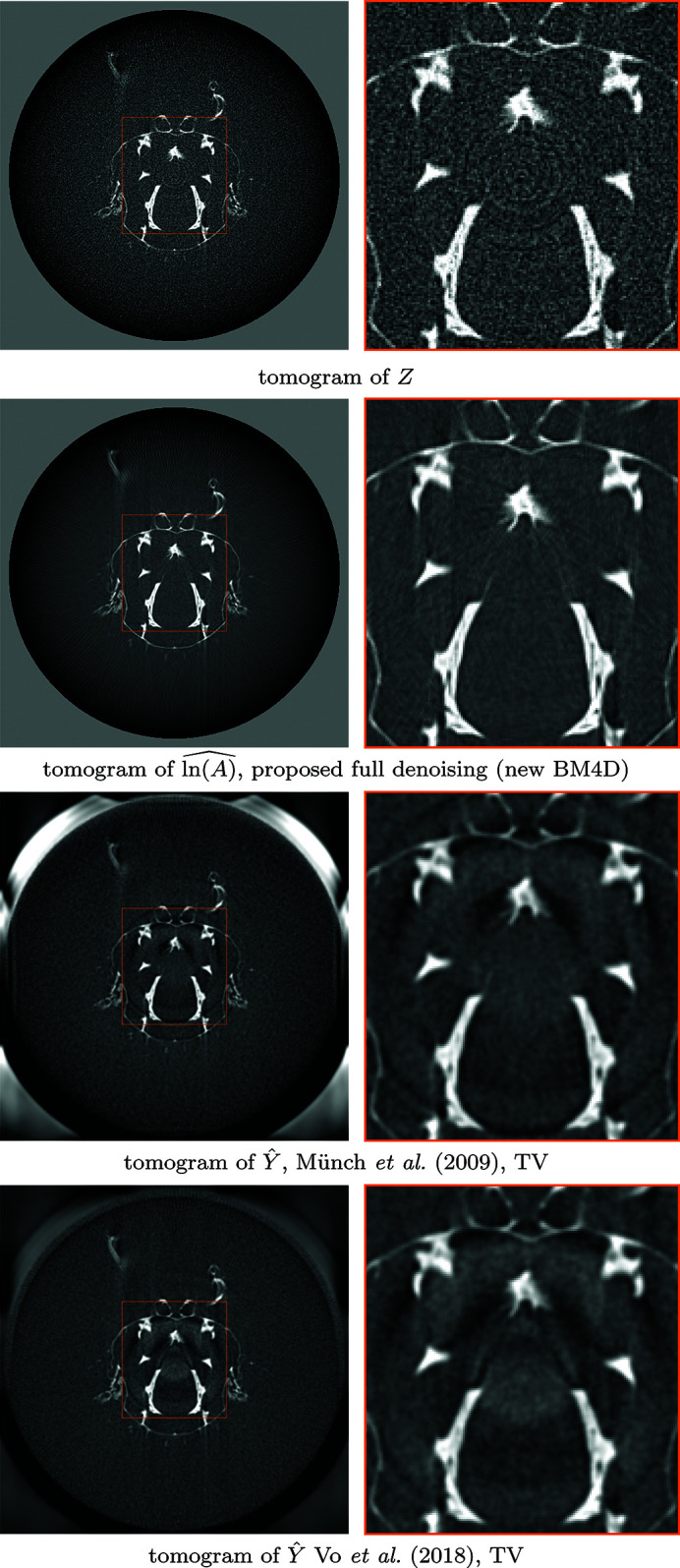
Comparison of fully denoised tomograms of *Fly*. Top-to-bottom: tomogram reconstructed from a noisy sinogram of the stack of projections *Z* (4), from the estimate of stack of underlying projections 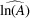 with proposed procedure with FBP reconstruction, and tomograms of the estimates for streak-free stacks 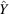 of Münch *et al.* (2009) ▸ with TV reconstruction, and 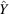 of Vo *et al.* (2018) ▸ with TV reconstruction. TV regularization was tuned visually, balancing residual noise and smoothing of signal. Compared with the reference methods, the proposed procedure manages to remove most noise without creating shadowlike artifacts common to Münch *et al.* (2009) ▸ and Vo *et al.* (2018) ▸.

The *Fly* dataset consists of 180 projections with 50 s exposure (detector pixel size 27 µm, demagnified to 15.7 µm by cone-beam geometry) collected using a Sigray Prisma X-ray micro-tomography instrument at 34 kV; the detector size is 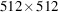 pixels. The denoising results for two different sinograms, as well as a corresponding tomogram after streak attenuation, are shown in Fig. 6 ▸. A comparison of denoising on a vertical slice of the stack of tomograms is shown in Fig. 7 ▸, and a comparison for fully denoised reconstructions is shown in Fig. 8 ▸.

**Figure 9:**
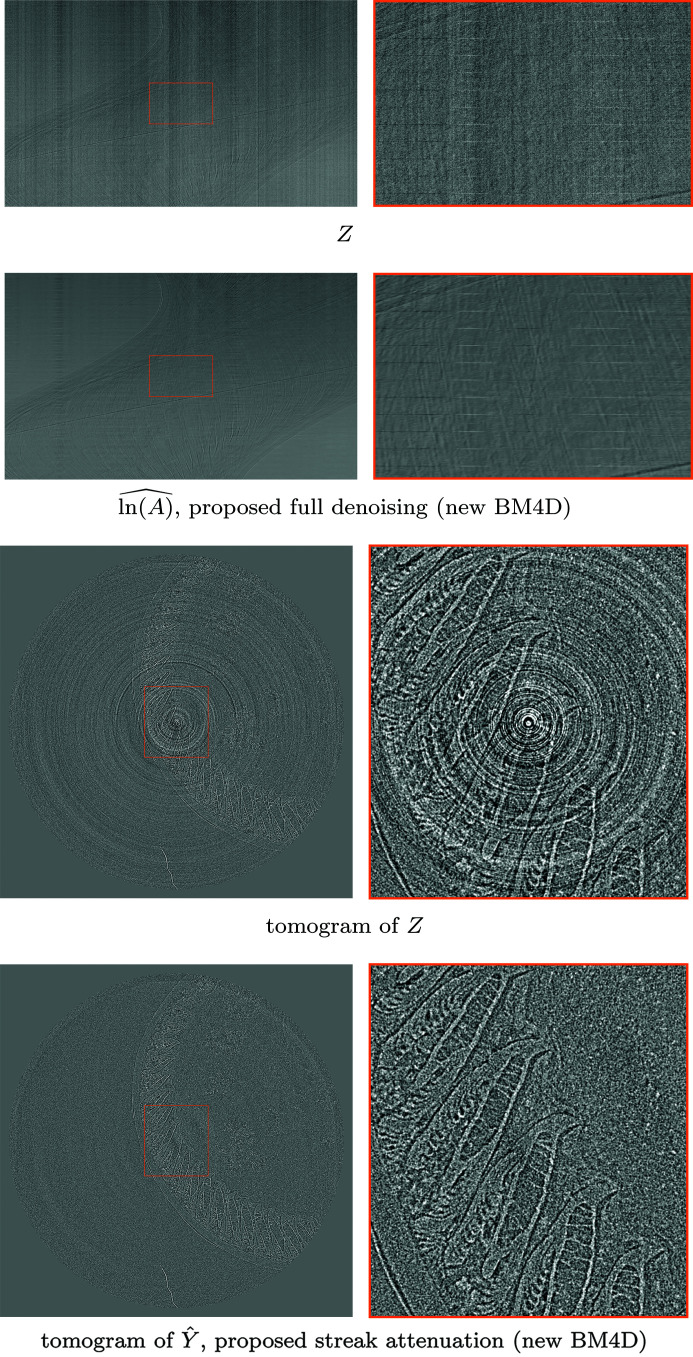
Denoising of the stack of projections of 00072. Top-to-bottom: display of a single sinogram of the noisy stack of projections *Z* (4), the corresponding estimate of the underlying projections 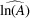 from the proposed procedure, and the corresponding tomograms of *Z* and the estimates 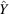 of streak-free stacks *Y* (5), respectively, obtained with filtered back-projection. The tomogram for 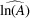 is shown in Fig. 11 ▸. Although the data present challenges through inconsistent noise intensities across the angular dimension, most streak noise and Poissonian noise is attenuated without notable loss of signal.

**Figure 10:**
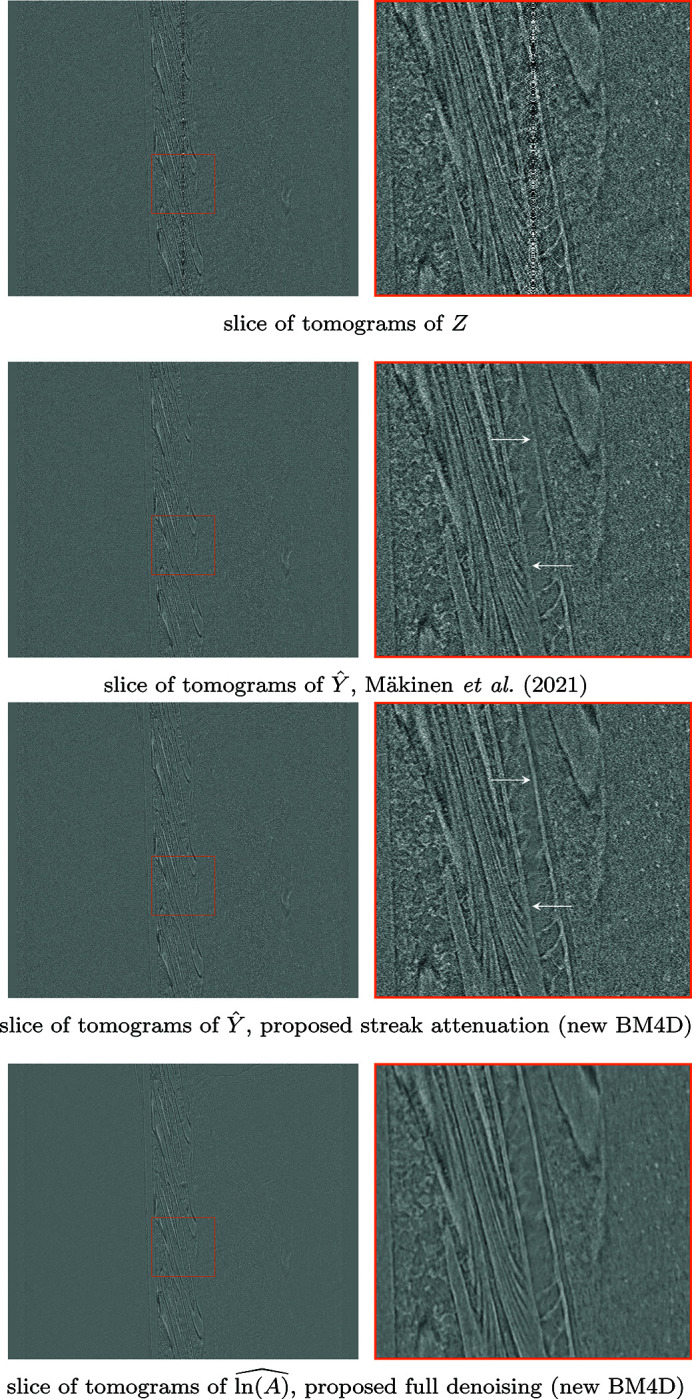
Comparison of central slices of resulting tomograms after different denoising procedures on 00072, where individual tomograms are horizontal lines each obtained with filtered back-projection. Displayed are slices of tomograms, reconstructed from, top-to-bottom: noisy projections *Z* (4), estimates 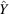 of the streak-free projections *Y* (5) denoised with Mäkinen *et al.* (2021) ▸, 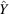 from the proposed streak denoising, and the result of the proposed full denoising 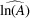 . Note the loss of signal near the central part of the Mäkinen *et al.* (2021) ▸ estimate, not observed in the proposed results.

**Figure 11:**
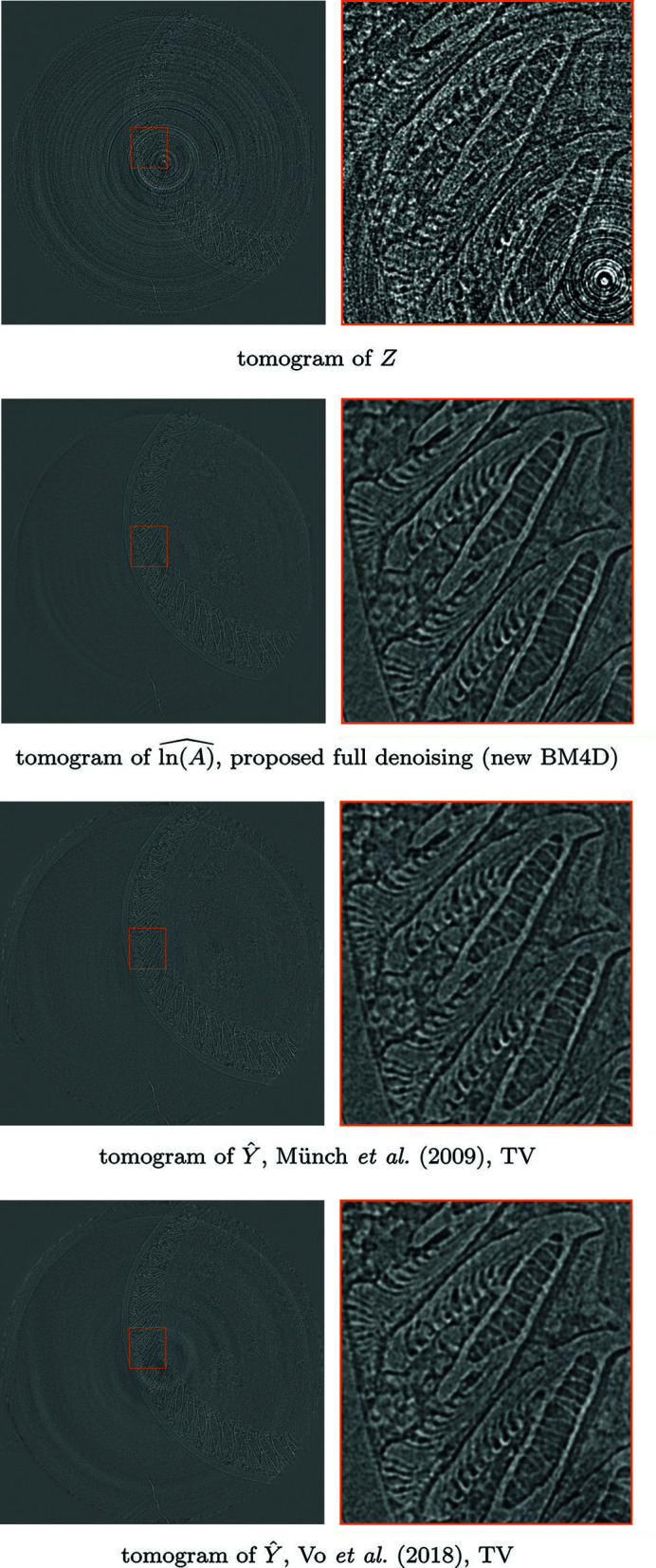
Comparison of fully denoised tomograms of 00072, corresponding to the sinogram in Fig. 9 ▸. Top-to-bottom: tomogram reconstructed from a noisy sinogram of the stack of projections *Z* (4), of the estimate of stack of underlying projections 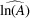 with the proposed procedure with FBP reconstruction, and tomograms of the estimates for streak-free stacks 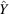 of Münch *et al.* (2009) ▸ with TV reconstruction, and 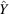 of Vo *et al.* (2018) ▸ with TV reconstruction. TV regularization was tuned visually, balancing residual noise and smoothing of signal. Compared with the reference methods, the proposed procedure manages to remove most streaks without significant loss of detail, as well as most Poissonian noise without excess smoothing of the signal.

We also test the algorithm on a soft tissue sample 00072 displaying severe ring artifacts freely available in TomoBank (De Carlo *et al.*, 2018 ▸). The data contain 1500 projections with 1.43 µm pixels, obtained at the Advanced Photon Source, 2-BM beamline; other experimental parameters are X-ray energy of 20 keV, 10 µm LuAG scintillator, and sample-to-detector distance of 15 mm. The detector size is 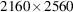 pixels. Included are ten samples for bright- and dark-fields, which are averaged to obtain a single bright-field and dark-field. A sinogram and a corresponding tomogram from the denoising results for streak removal are shown in Fig. 9 ▸, and slices of the stack of tomograms are compared in Fig. 10 ▸. Reconstructions of fully denoised projections are further compared in Fig. 11 ▸.

The proposed method achieves superior SNR values in streak removal in all simulated noise experiments. Although the difference to the 2-D implementation of Mäkinen *et al.* (2021) ▸ is not immediately visually obvious from individual sinograms or tomograms, the displayed vertical slices of the reconstructed objects show clear improvement in both signal preservation and avoiding discontinuity between different tomograms. Compared with Münch *et al.* (2009) ▸ and Vo *et al.* (2018) ▸, the proposed method avoids creation of shadow artifacts around strong signal features. Furthermore, performing the Poisson denoising through the proposed framework allows application of standard filtered back-projection reconstruction to data originally corrupted by Poisson noise, but can also improve quality of iterative TV reconstruction.

### 6.1. Parameters

For streak attenuation, we calculate *K* following the formula of horizontal binning from Mäkinen *et al.* (2021) ▸, using as the base the size of the smallest displacement dimension. As a result, we use 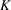 = 5 for 00072, 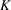 = 3 for *Fly*, and 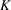 = 2 for the phantom. These values were found to offer a reasonable compromise between denoising wide streaks versus preserving low-frequency signal components. Other processing parameters are adjusted for the smaller block size and processing neighborhood of BM4D. For angular binning, we use 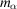 = 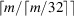 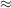 32 pixels, where *m* is the original angular size and *m*_α_ the output size; the resulting size is half of that used by Mäkinen *et al.* (2021) ▸. For segmentation of the streak denoising, we use a window of size 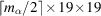 pixels. For the Poisson denoising, we use 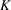 = 1 and 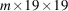 segments. For variance stabilization, we use the implementation ClipPoisGaus (Azzari & Foi, 2015 ▸) of Foi (2009 ▸) and Azzari & Foi (2014 ▸), and use a quadratic polynomial for the variance model *F*.

## 7. Discussion and conclusions

We have presented a framework for three-dimensional attenuation of streak noise extending the 2-D framework of Mäkinen *et al.* (2021) ▸, as well as a BM4D denoiser utilizing the algorithmic improvements of Mäkinen *et al.* (2020) ▸. Furthermore, we have included a denoising step for Poisson noise in the sinogram domain through BM4D and the adaptive variance stabilization of Foi (2009 ▸) and Azzari & Foi (2014 ▸).

We test the algorithm on both synthetic and real data, demonstrating superior SNR compared with other popular streak removal algorithms, and showing improvements in streak attenuation over Mäkinen *et al.* (2020) ▸. Furthermore, we compare the results with those which use the conventional BM4D for correlated noise, demonstrating that the included improvements in BM4D for correlated noise are essential for successful streak attenuation. The included Poisson denoising allows for full sinogram-domain denoising within the framework. By operating fully in the 3-D stack of projections, the 3-D structure of the data can be leveraged for more accurate noise removal. The proposed procedure is fully automatic and does not require extra input parameters.

To compare different methods under their own ideal conditions, we have specifically selected the TV regularization parameter values that provide the best reconstruction quality. However, in real-world applications, these values cannot be identified precisely, and too small or too large parameter values may lead to residual noise or excess smoothing of the reconstructions. Inclusion of the proposed Poisson denoising step allows for weaker regularization, but notably also reduces the effects of relative shifts in the parameter values, meaning that the reconstruction can be safely deployed even when the regularization cannot be precisely tuned.

To consider the computational cost, we note that both denoising steps of *Fly* ( 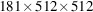 pixels) run single-threaded on an AMD Ryzen 7 1700 processor each take about one hour. The computational cost is mostly due to the BM4D denoising in CPU. Although the adopted implementation is single-threaded, the algorithm is embarrassingly parallel, and thus a highly parallel GPU-based implementation is expected to reduce the total run time to the scale of seconds (Davy & Ehret, 2020 ▸).

The Poissonian noise attenuation can also be performed without the preceding ring reduction step on data which do not display ring artifacts. In such case, 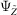 should be replaced by a flat PSD, as the Poissonian noise is approximately white prior to streak attenuation, whereas (28) considers the streak noise frequencies removed. Running the full denoising procedure in the absence of either streak or Poisson noise will lead to very small estimates for the corresponding noise components, meaning that no significant denoising will be performed for that noise.

We note that although we have focused on the full denoising of the projections, typical reconstruction pipelines, such as the iterative TV, provide further noise attentuation. For best results in combining the proposed denoising procedure with such pipelines, it may be necessary to adjust the filter strength for the denoising of Poissonian noise, *e.g.* for reduced attenuation of high-frequency noise, as it is further attenuated within the reconstruction. Likewise, integration of the proposed procedure within an iterative alternating reconstruction is left for future study.

## Figures and Tables

**Figure 5:**
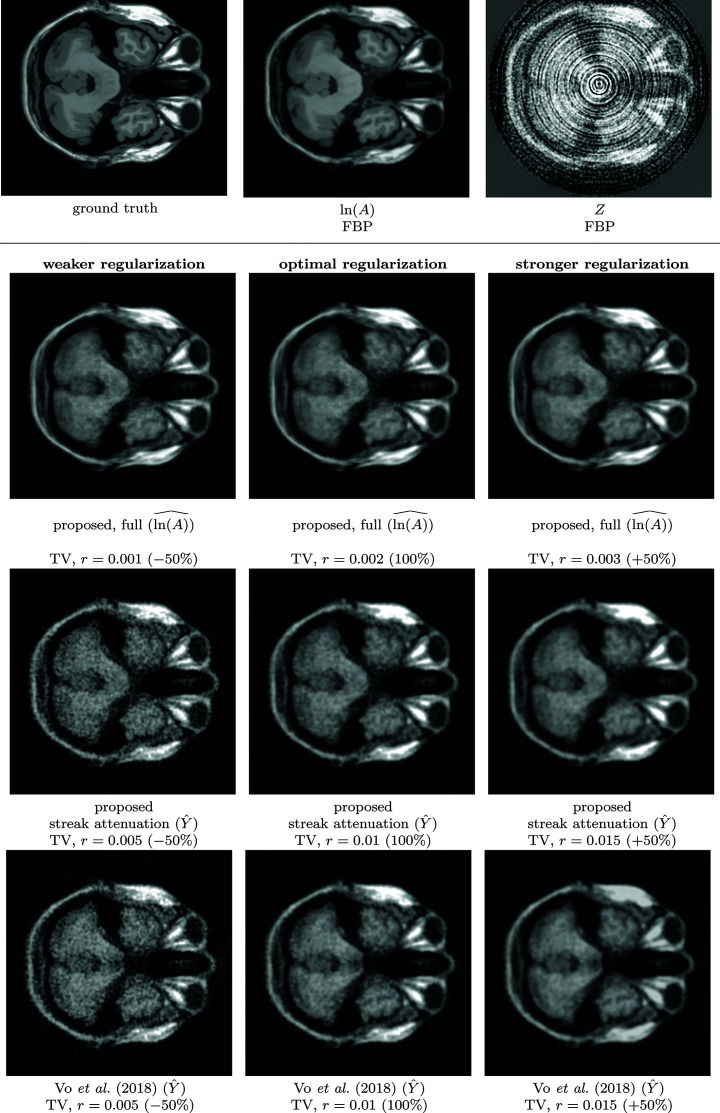
Comparison of reconstructions of the 3-D BrainWeb phantom corrupted with streak and Poisson noise as in (3), corresponding to the sinograms shown in Fig. 4 ▸. Top: ground truth volume, and reconstructions of ln(*A*) (3) and *Z* (4) obtained through filtered back-projection. Bottom: comparison of TV reconstruction of estimates with various regularization strengths *r*, where the percentage implies a multiplier to the regularization optimized to maximize SNR, *i.e.* 100% means ‘SNR-optimal’ regularization. Top-to-bottom: proposed full estimate ln(*A*), proposed streak-free estimate 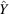 , and streak-free estimate of 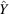 (Vo *et al.*, 2018) ▸, each with 100%, 50%, and 150% relative regularization strengths. Proposed estimates are computed embedding the improved BM4D. Notably, the full filtering offers improved reconstruction quality, and is also less sensitive to variations in the regularization parameters.

**Table 1:** Average signal-to-noise ratio for attenuation of streaks in the BrainWeb phantom subject to mixed streak and Poissonian noise as in (3), with different combinations of 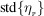 and peak values of *A*, with ‘peak’ = ∞ being the limiting case for which π = 0 Left-to-right: noisy stack of projections *Z* (4), and estimates 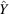 of the stacks of projections *Y* (5) denoised by the proposed procedure (12) (‘new BM4D’), proposed procedure embedding BM4D of Maggioni *et al.* (2012) ▸ (‘old BM4D’), Mäkinen *et al.* (2021) ▸, Münch *et al.* (2009) ▸, and Vo *et al.* (2018) ▸. As the table compares only streak removal, the SNR values are calculated with respect to the streak-free yet noisy projections 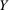 = 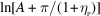 as 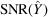 = 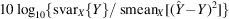 , where svar and smean denote sample variance and sample mean, respectively. Each value of the table is the average SNR over 10 different noise realizations.

Peak	std{η_P_}	*Z*	Proposed (new BM4D)	Proposed (old BM4D)	Mäkinen *et al.*(2021 ▸)	Munch *et al.* (2008 ▸)	Vo *et al.* (2018 ▸)
∞ (π = 0)	0.005	27.80	35.10	32.53	33.70	11.28	26.30
	0.01	21.85	32.60	28.97	29.66	11.26	25.33
	0.02	16.06	29.40	23.40	24.96	11.17	23.10
	0.05	8.82	24.44	14.64	18.22	10.61	18.53
5120	0.005	27.87	32.88	28.80	32.19	11.11	26.65
	0.01	21.91	31.00	26.91	29.00	11.09	25.49
	0.02	16.09	28.38	22.87	24.74	11.00	23.16
	0.05	8.83	24.04	14.64	18.25	10.47	18.55
2560	0.005	27.93	31.63	27.23	31.06	10.96	26.58
	0.01	21.97	30.03	25.55	28.38	10.94	25.36
	0.02	16.13	27.71	22.40	24.49	10.86	23.07
	0.05	8.85	23.69	14.63	18.23	10.34	18.54
1280	0.005	28.07	30.04	25.66	29.52	10.71	26.21
	0.01	22.09	28.72	23.79	27.44	10.69	25.03
	0.02	16.22	26.77	21.57	24.09	10.62	22.85
	0.05	8.90	23.16	14.61	18.21	10.15	18.49

**Table 2:** Average SNR for the reconstructed volumes of the BrainWeb phantom for the set of experiments shown in Table 1 ▸ Each reconstruction is performed with TV, either on the estimate 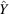 of the streak-free projections *Y* (5), or the estimate 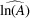 of the underlying stack of projections ln(*A*) (4). Left-to-right: reconstructed volumes of estimates produced by the proposed full procedure with improved BM4D, proposed streak removal with improved BM4D, proposed full procedure with old BM4D, Mäkinen *et al.* (2021) ▸, Münch *et al.* (2009) ▸, and Vo *et al.* (2018) ▸. Each regularization parameter *r* of TV is optimized individually for best SNR for each realization and method. The SNR values are each computed against the ground-truth noise-free phantom.

		Proposed (new BM4D)	Proposed (new BM4D)	Proposed (old BM4D)	Mäkinen *et al.* (2021 ▸)	Munch *et al.* (2008 ▸)	Vo *et al.* (2018 ▸)
Peak	std{η_P_}	TV 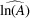	TV 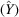	TV 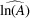	TV 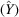	TV 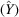	TV 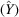
∞ (π = 0)	0.005	23.15	23.15	19.59	20.26	8.32	15.89
	0.01	21.05	21.05	17.09	18.01	8.25	15.39
	0.02	18.38	18.56	13.73	15.23	8.27	14.39
	0.05	14.23	14.75	11.29	11.05	8.04	12.11
5120	0.005	18.02	16.37	16.75	15.99	8.11	14.80
	0.01	17.45	16.00	15.70	15.41	8.10	14.49
	0.02	16.47	15.28	13.95	14.10	8.08	13.78
	0.05	14.03	13.41	11.02	10.82	7.92	11.87
2560	0.005	17.15	15.30	16.14	14.97	7.99	14.12
	0.01	16.75	15.03	15.21	14.53	7.99	13.82
	0.02	15.92	14.46	13.82	13.45	7.97	13.23
	0.05	13.76	12.85	10.94	10.67	7.87	11.67
1280	0.005	16.24	14.32	15.23	14.01	7.90	13.62
	0.01	15.95	14.13	14.71	13.71	7.90	13.40
	0.02	15.28	13.70	13.59	12.90	7.88	12.90
	0.05	13.41	12.43	10.87	10.50	7.77	11.43

## Footnotes

1 Despite its name, BM3D is a filter for 2-D images, where the third dimension originates from the nonlocal prior leveraged by the algorithm.

2 As *P*_raw_ is a 3-D array, the pixels of *I*_B_ and *I*_D_ are replicated through the angle dimension for the operations in (1).

3 Extreme streak noise arising from defective detectors is addressed separately in Section 4.2.

4 For the purpose of visualization and objective SNR comparison, we have corrected the intensity response of every estimate through a cubic polynomial such that it matches the ground truth. This is done particularly to improve the results of those methods such as Münch *et al.* (2009) ▸ whose intensity response significantly deviates from the ground truth.

## Appendix A Collaborative filtering and the BM4D denoising algorithm

### A1. Collaborative filtering

The rationale of transform-domain filtering is to work with a representation of the signal where most of the signal is compacted to only a few coefficients, whereas the remaining coefficients mostly comprise noise. Hence, by attenuating the coefficients with a non-linear shrinkage operator, it is possible to attenuate noise while keeping most of the signal intact. Nonlocal collaborative filters utilize this property in the context of collective transform coefficients of groups of similar patches extracted from the input. One of the most popular collaborative filters is the Block-Matching and 3-D filtering (BM3D) (Dabov *et al.*, 2007 ▸) denoising algorithm, which performs denoising on groups of blocks extracted from a 2-D image. In the BM4D volumetric denoiser (Maggioni *et al.*, 2012 ▸), the patches are 3-D volumes extracted from the volumetric data.

All operations of collaborative filters are made with regard to a reference patch moving through the volume. For each position of the reference patch, the following steps are executed:

(1) Collect similar patches into a group through **patch-matching**.

(2) Obtain a group transform spectrum by collectively transforming the group of patches.

(3) Perform **shrinkage**.

(4) Transform the shrunk spectra back to patch estimates and **aggregate** them to the original locations from which they were collected.

For details about the algorithm in arbitrary dimensionality, we refer the reader to Mäkinen *et al.* (2020) ▸. In the following section, we describe special considerations for the implementation of the algorithmic improvements in Mäkinen *et al.* (2020) ▸ for volumetric denoising.

### A2. Improved BM4D for correlated noise

Most of the improvements described by Mäkinen *et al.* (2020) ▸ are directly applicable to the 3-D denoiser. In this section, we consider extensions which are not immediate from the inclusion of an extra dimension.

#### A2.1. Shrinkage parameters

For the selection of shrinkage parameters λ and μ^2^, we embed the parameter selection subroutine of Mäkinen *et al.* (2020) ▸, which is based on the 2-D input PSD; for simplicity, we adopt directly also the pre-computed features and parameters computed for a set of 2-D PSDs. To utilize this system with a 3-D PSD, we include a simple procedure which obtains a 2-D projection of the 3-D PSD by preserving the two largest principal components, aiming to preserve the characterizing features of the PSD shape. This projection is then used to compute features as described for a 2-D PSD by Mäkinen *et al.* (2020) ▸ for the estimation of suitable λ and μ^2^.

#### A2.2. Fast implementation

We consider the fast implementation as suggested by Mäkinen *et al.* (2020) ▸. In particular, we perform all operations on a downscaled PSD of size  and compute exactly only the *K*_*f*_ first volumes of the 4-D spectrum and approximate the rest using the conventional variances. Furthermore, Fourier symmetries and sparsity of the transformed arrays can be exploited to reduce computational cost similar to the 2-D case.

#### A2.3. Refiltering

As noted by Mäkinen *et al.* (2020) ▸, even with exact modeling of the collaborative transform-domain noise spectrum, the accuracy of collaborative filtering is limited by the systemic factors arising from the used transforms, both in size and possible symmetries of the transform spectrum which may limit the modeling of the global PSD. As a result, the denoising may attenuate excess signal, leading to oversmoothing in some frequencies; Mäkinen *et al.* (2020) ▸ proposes the mitigation of these systemic issues through an extra filtering step performed on the denoising residual. The three-dimensional spectra are not exempt from these limitations, and as such we adopt the global Fourier thresholding and refiltering procedure through a 3-D FFT.
